# E protein binding at the *Tcra* enhancer promotes *Tcra* repertoire diversity

**DOI:** 10.3389/fimmu.2023.1188738

**Published:** 2023-07-06

**Authors:** Ariana Mihai, Sumedha Roy, Michael S. Krangel, Yuan Zhuang

**Affiliations:** Department of Immunology, Duke University School of Medicine, Durham, NC, United States

**Keywords:** E proteins, Tcra enhancer, V(D)J recombination, T cells, Thymus

## Abstract

V(D)J recombination of antigen receptor loci is a highly developmentally regulated process. During T lymphocyte development, recombination of the *Tcra* gene occurs in CD4^+^CD8^+^ double positive (DP) thymocytes and requires the *Tcra* enhancer (Eα). E proteins are known regulators of DP thymocyte development and have three identified binding sites in Eα. To understand the contribution of E proteins to Eα function, mutants lacking one or two of the respective binding sites were generated. The double-binding site mutant displayed a partial block at the positive selection stage of αβ T cell development. Further investigation revealed loss of germline transcription within the *Tcra* locus at the Jα array, along with dysregulated primary and impaired secondary Vα-Jα rearrangement. Eα E protein binding increases *Tcra* locus accessibility and regulates TCRα recombination, thus directly promoting *Tcra* repertoire diversity.

## 1 Introduction

The adaptive immune system recognizes a wide variety of antigens by way of B cell receptors and T cell receptors (TCRs) on the surfaces of B and T cells, respectively. These highly diverse antigen receptors (AgRs) are generated by the process of V(D)J recombination during defined stages of B and T lymphocyte development (1). V(D)J recombination is catalyzed by the lymphoid-specific recombination activating gene (RAG) proteins, which act on AgR loci to create double-strand breaks between variable (V), diversity (D), and joining (J) gene segments and their respective recombination signal sequences. AgR diversity depends on combinatorial usage of V, D, and J segments, together with junctional heterogeneity introduced by non-homologous end joining DNA repair mechanisms (1, 2).

V(D)J recombination occurs in an ordered fashion during B and T cell development due to the activity of developmentally regulated enhancer and promoter elements (1). These elements drive germline transcription and chromatin accessibility at defined sites in AgR loci, which are then permissive for RAG binding. RAG proteins generally assemble on highly accessible D and J gene segment recombination signal sequences to form a chromatin structure referred to as the recombination center (RC). RC-bound RAG is then able to capture V gene segments to complete the recombination process (1, 3).

Fetal liver or bone marrow origin precursors that migrate to the thymus can commit to the T cell fate and further differentiate into one of several mature T cell lineages. CD4^-^CD8^-^ double negative (DN) thymocytes rearrange the *Tcrg*, *Tcrd*, and *Tcrb* genes, leading to TCRγ/TCRδ or TCRβ/pre-Tα (pre-TCR) pairings. This allows adoption of the γδ or αβ T cell lineage fates, respectively (4). Having passed the β-selection checkpoint, developing αβ T cells proceed to the CD4^+^CD8^+^ double positive (DP) stage and *Tcra* rearrangement. Uniquely, *Tcrd* and *Tcra* gene segments are arranged in a single genetic locus. In the mouse, the *Tcra-Tcrd* locus is organized as an array of about 100 V gene segments, followed by Dδ, Jδ, and Cδ gene segments, 60 Jα gene segments, and lastly Cα (5). This configuration leads to deletion of *Tcrd* upon *Tcra* rearrangement. *Tcra* rearrangement requires the *Tcra* gene enhancer (Eα), located directly downstream of Cα (6). Lymphoid specific transcription factors (TFs) bind adjacent to or within four defined protein-binding elements (Tα1-Tα4) that make up Eα (7–9). These TFs include c-MYB, RUNX1, RUNX3, GATA3, ETS1/FLI1, SP1, CREB, LEF1/TCF1, CTCF, E2A/HEB, NFAT, AP1, and EGR1, with some having more than one identified binding site with the 275-bp element (10). TF occupancy and histone modification is observed as early as the DN stage, though Eα activation only occurs after pre-TCR signaling (11). Eα acts in *cis* to activate locus germline transcription, assemble an RC, and initiate Vα-Jα rearrangement. Initially, Eα activates the T early alpha (TEA) and *Traj*49 promoters associated with the most Vα-proximal Jα gene segments (12–14). The assembled RC then directs an initial round of Vα-Jα rearrangement, referred to as a primary rearrangement, to nearby Jα segments. However, owing to the lack of D segments, *Tcra* is capable of undergoing multiple rounds of Vα-Jα rearrangement, and these secondary rearrangements will continue until a TCR is created that can mediate positive selection. Secondary rearrangements are thought to depend on RCs created by Eα and the promoter of the rearranged V gene segment (15). Following positive selection, Eα activity is downregulated in single-positive thymocytes and mature αβ T lymphocytes (16).

E proteins are a class of basic helix-loop-helix (bHLH) proteins that recognize a canonical CANNTG DNA sequence, also referred to as an E-box (17). In developing T cells, the E2A (*Tcf3*) and HEB (*Tcf12*) E proteins have wide-ranging targets including genes regulating cell survival and cell-cycle progression, control of developmental checkpoints, and stage-specific chemokine expression (17). E proteins are important regulators of V(D)J recombination. In both developing B and T cells, E proteins regulate the stage-specific expression of the RAG genes (18, 19). E proteins also regulate germline transcription at the *Igh*, *Igk*, *Igl*, *Tcrg*, *Tcrd*, and *Tcrb* genes leading to recombination permissive chromatin environments (17, 20–22). Furthermore, E proteins are essential regulators of T cell development checkpoints, and have been previously shown to control DP cell development and transition to the SP stage (23–26). Of the multiple TFs binding at Eα, E proteins are notable in that they occupy three identified binding sites during thymocyte development and show diminished binding in mature αβ T lymphocytes (11, 16, 27, 28). However, it is not known whether E proteins have direct effects on Eα activity and *Tcra* recombination, in part due to the broad impacts, noted above, on the DP thymocyte population in mouse E protein knockout models.

To assess the role of E proteins in *Tcra* rearrangement, E-boxes at Eα were deleted. Loss of the 5′ E-box (E1) had no discernable effect on thymocyte development. However, the additional deletion of the 3′ E-box (E3) impaired positive selection of DP thymocytes, with a concordant loss of CD4^+^ and CD8^+^ single positive (SP) thymocytes. The double E-box deletion reduced germline transcription across Jα segments, which is expected to result in reduced accessibility for Vα-Jα recombination. Consistent with this, alterations in Jα segment usage and invariant natural killer T (iNKT) cell development were detected, suggestive of dysregulated primary and impaired secondary Vα-Jα rearrangement. Therefore, E protein binding to Eα increases *Tcra* locus germline transcription, regulates Jα segment recombination, and promotes *Tcra* repertoire diversity.

## 2 Materials and methods

### 2.1 Mice

The ΔE1 mutation was generated in B6SJLF1/J (RRID : IMSR_JAX:100012). E1 was targeted with a two-guide and donor (**Supplementary Table 1**) CRISPR/Cas9 approach by pro-nuclear injection. Founders were screened by Sanger sequencing; identified mutants lacked the donor sequence. Founders were then crossed to lab-maintained C57BL/6. Eα^ΔE1/ΔE1^ pups from Eα^ΔE1/+^ by Eα^ΔE1/+^ breeding were screened by Sanger sequencing for C57BL/6 and SJL/J polymorphisms within *Tcra-Tcrd*, all matching to the C57BL/6 reference sequence (**Supplementary Table 1**).

Single-guide (**Supplementary Table 1**) CRISPR/Cas9 electroporation of Eα^ΔE1/ΔE1^ was used to generate alleles with two mutated Eα E-boxes. Founders were screen by Sanger sequencing. The ΔE1ΔE3 and ΔE1ΔE3(1) alleles were detected in different founders and maintained separately by crossing to lab-maintained C57BL/6. The mutation was introduced onto a RAG-deficient background by crossing ΔE1ΔE3 to *Rag1^tm1Mom^*/J (RRID : IMSR_JAX:002216).

Except where noted, all analyses were performed on mice 3-4 weeks of age obtained from heterozygous by heterozygous Eα allele breeding. All mice were of mixed C57BL/6 and SJL/J strain background.

The Duke University Cancer Institute Transgenic and Knockout Mouse Shared Resource carried out the above CRISPR/Cas9-mediated mutagenesis. All mice were bred in a Duke University Division of Laboratory Animal Resources specific pathogen-free facility and handled in accordance with protocols approved by the Duke University Institutional Animal Care and Use Committee.

### 2.2 Antibodies

Fluorescently conjugated antibodies used in flow cytometry and cell sorting are commercially available and have been previously validated (**Supplementary Table 2**). CD1d tetramer was obtained from the National Institutes of Health Tetramer Core Facility.

### 2.3 Flow cytometry and cell sorting

Thymus was harvested and dissociated to single-cell suspension in FACS buffer (2.5% FBS and 2 mM EDTA supplemented PBS) and filtered using 70 nm nylon mesh. For analysis by flow cytometry, 3 x 10^6^ cells were stained with fluorescently labeled antibodies and loaded CD1d-tetramer for 30 minutes at 4°C and then washed with excess FACS buffer. Samples were re-suspended in FACS buffer containing 7-Aminoactinomycin D (7-AAD) (ThermoFisher Scientific, Cat. A1310) or DAPI (Sigma-Aldrich, Cat. D9542) and analyzed on FACSCantoII or Fortessa X20 (BD Biosciences) cytometers available via the Duke University Cancer Institute Flow Cytometry Shared Resource. Analysis was performed using FlowJo (version 10.8.1) software. Gating scheme is shown in **Supplementary Figure 1**.

For isolation of preselection thymocytes, sorting was performed using an Astrios (Beckman-Coulter) cell sorter by the Duke University Flow Cytometry Shared Resource. Sample staining included a lineage dump (B220, CD11b, CD11c, CD19, GR-1, TER119, F4/80, and TCRδ antibodies). Gating scheme is shown in **Supplementary Figure 2**.

### 2.4 *Tcra* repertoire library preparation

Three to five million pre-selection DP thymocytes (CD4^+^CD8^+^CD3^lo^) were sorted from Eα^+/+^ and Eα^ΔE1ΔE3/ΔE1ΔE3^ thymuses. Sorted cells were re-suspended in TRIzol and stored at -80°C for later RNA extraction. RNA was purified using Direct-Zol RNA Microprep (Zymo Research) kit with on-column DNase digestion. Sequencing libraries were prepared with modification of previously published methods (29, 30). Briefly, 5 ng of total RNA was reverse transcribed using SmartScribe Reverse Transcriptase (Takara Bio, Cat. 639538) with Trac-RT and SMARTnnnA template switch oligo (**Supplementary Table 1**). Samples were then treated for 40 min at 37°C with 5 units uracil DNA glycosylase (NEB). cDNA purification was carried out using Ser-Mag Carboxylate-Modified Magnetic SpeedBeads (GE Healthcare Life Sciences). Q5 high-fidelity polymerase (NEB) was used to amplify cDNA (18 cycles), which was then purified using Ser-Mag Carboxylate-Modified Magnetic SpeedBeads. Q5 high-fidelity polymerase was used to perform dual-indexing of amplified cDNA, followed again by Ser-Mag Carboxylate-Modified Magnetic SpeedBeads purification. Sample quality control was performed by agarose gel electrophoresis. Replicate Eα^+/+^ and Eα^ΔE1ΔE3/ΔE1ΔE3^ indexed samples were pooled at equal concentration and final library preparation carried out using NEBNext Ultra II DNA library preparation kit (NEB). Library was gel extracted using Zymoclean Gel DNA recovery kit (Zymo Research). Sequencing was performed on a MiSeq sequencer (Illumina) (300 x 300 bp) by the Duke University Cancer Institute Sequencing and Genomic Technologies Shared Resource.

### 2.5 *Tcra* repertoire analysis

*Tcra* repertoire analysis followed previously published protocols (29, 30). The following were performed using Migec v1.2.9 (31) (RRID : SCR_016337) (1): demultiplexing of raw fastq files (function: Checkout; parameters: -cute); (2) assessment of molecular identifier group (MIG) size distribution (function: Histogram; parameters: default); collapse of UMIs and filtering (function: AssembleBatch; parameters: -force-collision-filter –force-overseq 5). Collapsed reads were merged using MiTools v1.5 (https://github.com/milaboratory/mitools) (function: merge; parameters: -I –s 0.7). MiXCR v3.0.13 (32) (RRID : SCR_018725) was used to perform: read alignment (function: align; parameters: -s mmu –OvParameters.geneFeatureToAlign=VTranscript); assemble clonotypes (function: assemble; parameters: -OassemblingFeatures=[CDR2+FR3+CDR3] –OclusteringFilter.specificMutationProbability=1E-4); clones export (function: exportClones; parameters: default). VDJTools v1.2.1 (33) was used to convert files appropriately (function: Convert; parameters: default) and determine segment usage (function: CalcSegmentUsage; parameters: -u).

### 2.6 Generation of RAG-deficient DP thymocytes

Experimental mice were injected intraperitoneally with 150 µg anti-CD3ϵ (BioLegend, Cat. 145-2C11) at 3 weeks of age. At 10 days post-injection, thymus was harvested and dissociated to single-cell suspension in FACS buffer. Whole thymocytes were re-suspended in TRIzol and stored at -80°C for later RNA extraction.

### 2.7 Reverse transcription

RNA was purified using Direct-Zol RNA Microprep (Zymo Research) kit with on-column DNase digestion. cDNA synthesis was performed using SuperScript III Reverse Transcriptase (Invitrogen, Cat. 108080093) with random hexamers as per manufacturer instructions.

### 2.8 RT-qPCR

Reverse transcription quantitative polymerase chain reaction (RT-qPCR) was performed with PowerTrack SYBR Green Master Mix (ThermoFisher Scientific, Cat. A46109) as per manufacturer instructions, using 5 ng of cDNA per 20 µL reaction. Reactions were carried out in Axygen PCR microplate (PCR-96-LP-FLT-C) with MicroAmp Optical Adhesive Film (ThermoFisher Scientific, Cat. 4311971) using an Eppendorf MasterCycler qPCR machine. Values shown were normalized to those for *Actb*. Primers are shown in **Supplementary Table 1**.

### 2.9 ChIP

Chromatin immunoprecipitation (ChIP) was adapted from a previously published protocol (34). Dynabeads M-280 Sheep anti-Mouse IgG (Invitrogen, Cat. 11202D) (100 μL per sample) were washed four times using PBS-BSA (1x PBS, 5 mg/mL BSA) (ThermoFisher Scientific, Cat. 15260037). Dynabeads were resuspended in PBS-BSA (250 μL per sample) with 8 μg (per sample) anti-E2A (Santa Cruz Biotechnology, Cat. sc-416X; Yae), anti-HEB (Santa Cruz Biotechnology, Cat. sc-28364X; D-3), anti-GATA3 (Santa Cruz Biotechnology, Cat. sc-268X; HG3-31), or anti-RUNX1 (Santa Cruz Biotechnology, Cat. sc-365644X; A-2), and incubated at 4°C for 24 hours with rotation. Dynabeads were washed three times with PBS-BSA, and aliquoted to sample tubes for final wash. Whole thymocytes were resuspended at 10 x 10^6^ cells/mL in 1% paraformaldehyde (ThermoFisher Scientific, Cat. 28906) in RPMI 1640 (ThermoFisher Scientific, Cat. 11875093) (supplemented with 10% FBS) for cross-linking. After 10 min at room temperature, cross-linking reaction was stopped by adding glycine to a final concentration of 0.125 M. Cross-linked cells were pelleted (at 4°C) and washed with 1x PBS (4°C). Cross-linked cells were resuspended at 20 x 10^6^ cells/mL in ChIP Lysis Buffer (Santa Cruz Biotechnology, Cat. sc-45000) and incubated 5 min on ice. Cross-linked cells were pelleted and resuspended at 10 x 10^6^ cells/mL in ChIP Lysis Buffer High Salt (Santa Cruz Biotechnology, Cat. sc-45001). Samples were sonicated for four cycles (30 s on, 30 s off) using a Bioruptor Pico (Diagenode). Samples were pelleted 15 min at 13200 rpm (4°C). From the supernatant, 1% was removed and stored at -20°C for use as input. Antibody-bound Dynabeads were resuspended in 500 μL of sample supernatant (5 x 10^6^ cells starting material) and incubated at 4°C for 16 hours with rotation. Beads were washed five times with ChIP Wash Buffer (Santa Cruz Biotechnology, Cat. sc-45002), with 3 min incubation at 4°C with rotation between washes. Beads were resuspended in 1 mL TE and rotated 1 min at room temperature. Supernatant was discarded and beads were resuspended in 200 μL ChIP Elution Buffer (Santa Cruz Biotechnology, Cat. sc-45003). Samples were incubated 1 hour at 65°C at 1500 rpm. Supernatant was transferred to new tube and incubated 16 hours at 65°C at 1500 rpm. 150 μL ChIP Elution Buffer was added to 50 μL input sample and incubated 16 hours at 65°C at 1500 rpm. To each sample 200 μL TE and 80 μg RNase A (ThermoFisher Scientific, Cat. EN0531) were added, and then incubated 1 hour at 37°C at 1000 rpm. To each sample 80 μg proteinase K (ThermoFisher Scientific, Cat. AM2546) was added, and then incubated 30 min at 56°C at 1000 rpm. DNA was purified by phenol/chloroform/ethanol extraction, and resuspended in 50 μL TE. Samples were assayed by qPCR with PowerTrack SYBR Green Master Mix as per manufacturer instructions, using 2 μL DNA per 20 µL reaction. Reactions were carried out in Axygen PCR microplate with MicroAmp Optical Adhesive Film using an Eppendorf MasterCycler qPCR machine. Values shown are relative to input. Primers are shown in **Supplementary Table 1**. PBS-BSA, ChIP Lysis Buffer, ChIP Lysis Buffer High Salt, and ChIP Wash Buffer contained protease inhibitors (Roche, Cat. 11697498001).

### 2.10 Statistical analysis

All reported data are from individual mice, with no repeated measurements from the same sample. Sample size was not predetermined by statistical methods. GraphPad Prism (version 9.5.0) software was used for all statistical analyses and generation of graphs.

## 3 Results

### 3.1 Normal αβ T cell development upon deletion of a single Eα E-box

**Figure 1:**
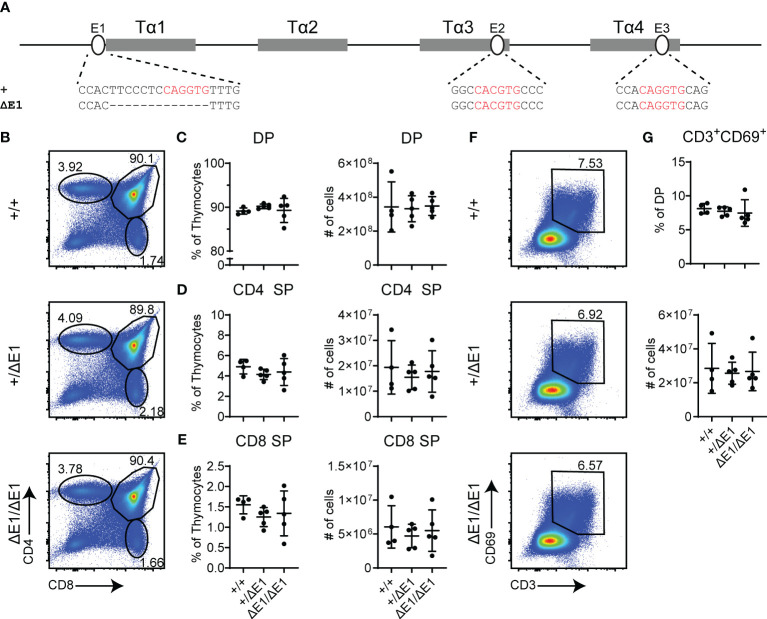
Loss of E1 does not impair T cell development. **(A)** Diagram of relative positions of Tα1-Tα4 protein binding regions and E1-E3 E protein binding sites within Eα, with sequences of wild-type (+) and ΔE1 mutation indicated below. E protein binding motifs are highlighted in red. **(B)** Representative flow cytometry plots, displayed as CD4 versus CD8, of live thymocytes from Eα^+/+^, Eα^+/ΔE1^, and Eα^ΔE1/ΔE1^ mice. Frequencies of gated populations are shown. **(C–E)** Frequencies and numbers of **(C)** DP thymocytes, **(D)** CD4^+^ SP thymocytes, and **(E)** CD8^+^ SP thymocytes, in Eα^+/+^, Eα^+/ΔE1^, and Eα^ΔE1/ΔE1^ mice, with gating as shown in **Supplementary Figure 1**. Note that CD8^+^ SP are gated as CD8^+^TCRβ^+^CD24^-^ to exclude CD8 immature single positives. **(F)** Representative flow cytometry plots, displayed as CD69 versus CD3, of DP thymocytes (gated as shown in **(B)**) from Eα^+/+^, Eα^+/ΔE1^, and Eα^ΔE1/ΔE1^ mice. Frequencies of gated populations are shown. **(G)** Frequency and number of CD3^+^CD69^+^ DP cells in Eα^+/+^, Eα^+/ΔE1^, and Eα^ΔE1/ΔE1^, following gating as shown in **(F)**. Data are pooled from 3 independent experiments and are plotted as mean ± SD. Eα^+/+^ (n = 4), Eα^+/ΔE1^ (n = 5), and Eα^ΔE1/ΔE1^ (n = 5). Statistical analysis: one-way ANOVA with correction for multiple comparison using Tukey’s *post hoc* testing. Significant differences were not detected.

To assess the impact of E protein binding at Eα, the E-box upstream of Tα1 (E1) was targeted by CRISPR/Cas9. The resulting 13 base pair (bp) deletion (ΔE1) eliminates the 6 bp E-box; although the upstream 7 bp were also removed and base pair changes occur at the gRNA recognition sequence (**Figure 1A**, **Supplementary Figure 3A**), no transcription factor binding was detected at these sites in previous dimethylsulfate genomic footprinting experiments (27). The generated allele is hereafter denoted as Eα^ΔE1^.

Eα deletion blocks thymocyte development at the DP stage, albeit with normal thymus cellularity (6). Using flow cytometry, no significant difference was detected in the numbers and proportions of total, DP, and SP thymocytes in the Eα^ΔE1/ΔE1^ mutants when compared to Eα^+/+^ littermates (**Figures 1B–E**, **Supplementary Figure 3B**). Further analysis of CD3 and CD69 expression did not show a significant difference in DP cells that are positively selected in Eα^ΔE1/ΔE1^ mice (**Figures 1F, G**).

### 3.2 Deletion of two Eα E-boxes impairs positive selection

**Figure 2:**
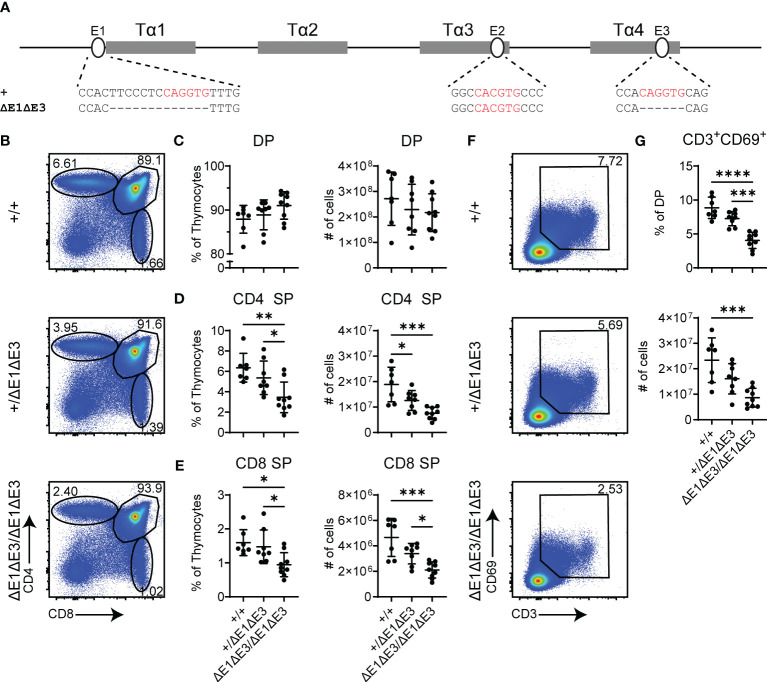
Loss of E1 and E3 impairs T cell development. **(A)** Diagram of relative positions of Tα1-Tα4 protein binding regions and E1-E3 E protein binding sites within Eα, with sequences of wild-type (+) and ΔE1ΔE3 mutation indicated below. E protein binding motifs are highlighted in red. **(B)** Representative flow cytometry plots, displayed as CD4 versus CD8, of live thymocytes from Eα^+/+^, Eα^+/ΔE1ΔE3^, and Eα^ΔE1ΔE3/ΔE1ΔE3^ mice. Frequencies of gated populations are shown. **(C–E)** Frequencies and numbers of **(C)** DP thymocytes, **(D)** CD4^+^ SP thymocytes, and **(E)** CD8^+^ SP thymocytes, in Eα^+/+^, Eα^+/ΔE1ΔE3^, and Eα^ΔE1ΔE3/ΔE1ΔE3^ mice. **(F)** Representative flow cytometry plots, displayed as CD69 versus CD3, of DP thymocytes [gated as shown in **(B)**] from Eα^+/+^, Eα^+/ΔE1ΔE3^, and Eα^ΔE1ΔE3/ΔE1ΔE3^ mice. Frequencies of gated populations are shown. **(G)** Frequencies and numbers of CD3^+^CD69^+^ DP thymocytes in Eα^+/+^, Eα^+/ΔE1ΔE3^, and Eα^ΔE1ΔE3/ΔE1ΔE3^, with gating as shown in **(F)**. Data are pooled from 4 independent experiments and are plotted as mean ± SD. Eα^+/+^ (n = 7), Eα^+/ΔE1ΔE3^ (n = 8), and Eα^ΔE1ΔE3/ΔE1ΔE3^ (n = 9). Statistical analysis: one-way ANOVA with correction for multiple comparison using Tukey’s *post hoc* testing. *p < 0.05, **p < 0.01, ***p < 0.001, ****p < 0.0001.

To further impact E protein binding at Eα, the E3 E-box (located in Tα4) was targeted for deletion in the Eα^ΔE1^ allele. The generated Eα^ΔE1ΔE3^ allele precisely eliminates the 6 bp E3 E-box (**Figure 2A**). A second allele, Eα^ΔE1ΔE3(1)^, disrupts E3 by a 1 bp deletion (**Supplementary Figure 4A**). As in Eα^ΔE1/ΔE1^, thymic cellularity in Eα^ΔE1ΔE3/ΔE1ΔE3^ double mutants did not differ from that of wild-type littermates (**Supplementary Figure 4B**). However, analysis by flow cytometry revealed clear reductions of CD4^+^ and CD8^+^ thymocytes in the double mutants (**Figures 2B–E**). This suggests reduced positive selection, a supposition confirmed by the substantial reductions in both the frequencies and numbers of CD3^+^CD69^+^ DP thymocytes (**Figures 2F, G**). The developmental block was similarly observed in Eα^ΔE1ΔE3(1)/ΔE1ΔE3(1)^ double mutants (**Supplementary Figures 4C–I**).

**Figure 3:**
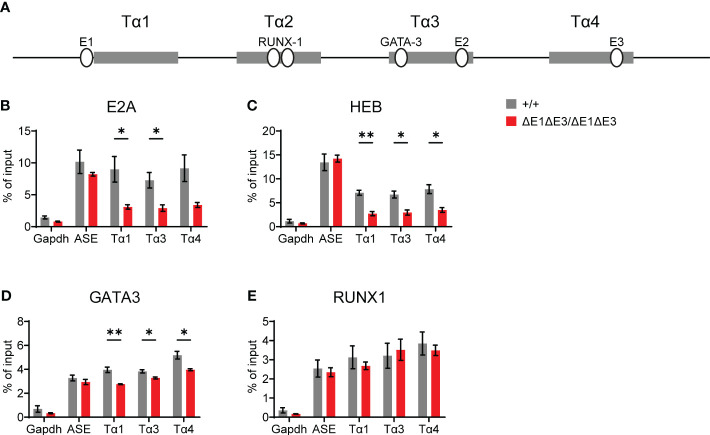
Loss of E1 and E3 reduces E protein and GATA3 binding at Eα. **(A)** Diagram of relative positions of Tα1-Tα4 protein binding regions and GATA3, RUNX1, and E1-E3 E protein binding sites within Eα. ChIP-qPCR of E2A **(B)**, HEB **(C)**, GATA3 **(D)**, and RUNX1 **(E)** binding in Eα^+/+^ (n = 3) and Eα^ΔE1ΔE3/ΔE1ΔE3^ (n = 3) whole thymocytes. The RAG ASE and *Gapdh* served as positive and negative controls, respectively. Data were pooled from 3 independent experiments, one of which included an age-matched C57BL/6 as Eα^+/+^. Data are plotted as mean ± SEM. Statistical analysis: Student’s t test. *p < 0.05, **p < 0.01.

To understand the molecular basis for this block, TF binding at Eα was assessed by ChIP-qPCR. As per expectations, E2A and HEB binding was significantly reduced (**Figures 3A–C**). That reductions were only partial and were detected at all three E protein binding sites likely reflects residual E protein binding at the intact E2 site (in Tα3), coupled with the inability of ChIP to resolve binding signals at E protein binding sites separate by no more than 150 bp. Interestingly, ablation of E-boxes in Tα1 and Tα4 provoked mildly reduced binding of GATA3 (whose defined binding site is in Tα3), but not RUNX1 (whose defined binding sites are in Tα2) (**Figures 3A, D, E**) (27). In contrast, no changes in transcription factor binding were detected at the RAG anti-silencer element (ASE), which contains binding sites for all of these factors. These results highlight that loss of E protein binding may mediate effects in part by destabilizing the binding of other components of the Eα enhanceosome, with some transcription factors having greater dependence on E-protein binding than others.

Although impaired, Eα likely retains considerable activity in double mutant mice given the continued progression of αβ T cell development and generation of SP thymocytes. No impact was observed on the development of γδ T cells (**Supplementary Figures 5A–C**).

### 3.3 Eα E-box deletion reduces *Tcra-Tcrd* germline transcription

**Figure 4:**
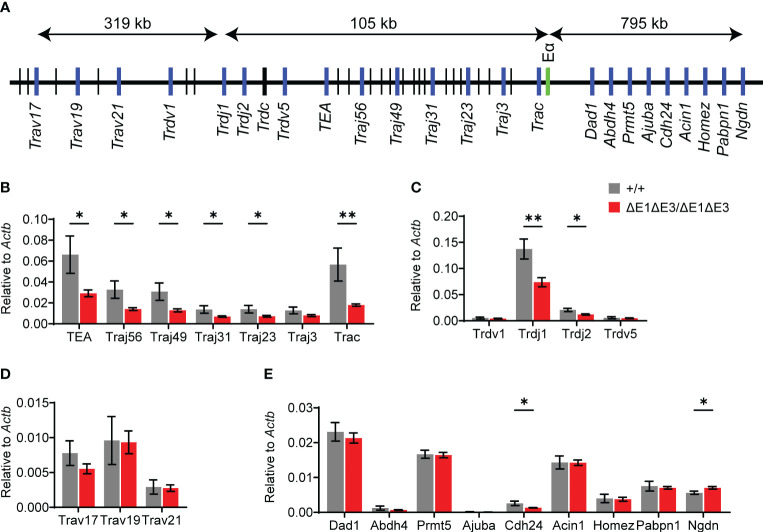
Loss of E protein binding at Eα reduces *Tcra-Tcrd* locus transcription. **(A)** Diagram of murine *Tcra-Tcrd* locus region assayed by RT-qPCR. Diagram is not to scale; distances between features are indicated. Relative position of assayed genes and gene segments (blue), Eα (green), and other gene segments (black) are shown. **(B–E)** RT-qPCR analysis of transcription in RAG-deficient Eα^+/+^ (n = 5) and Eα^ΔE1ΔE3/ΔE1ΔE3^ (n = 8) DP thymocytes. Data were pooled from 3 independent experiments. Data are plotted as mean ± SEM. Statistical analysis: Student’s t test. *p < 0.05, **p < 0.01.

Eα activity was evaluated by using RT-qPCR to assess transcription of known Eα targets in DP thymocytes from *Rag1*-deficient Eα^ΔE1ΔE3/ΔE1ΔE3^ and Eα^+/+^ littermates (**Figure 4A**). Eα acts in *cis* to activate the T early-α (TEA) promoter immediately 5′ of the Jα array, and the Jα49 promoter 15 kb downstream of TEA. These promoters target primary Vα-Jα rearrangements to the most proximal Jα segments, after which they are excised (12, 13, 35, 36). Jα accessibility in secondary rearrangements is driven by promoters of rearranged Vα segments (15). The double E-box mutant showed a significant loss of germline transcription for the region spanning TEA to Cα (*Trac*) (**Figure 4B**). Upstream of TEA, the Jδ segments showed loss of transcription while proximal Vα segments had no significant difference in expression (**Figures 4C, D**). Expression of genes downstream of *Tcra-Tcrd* likewise remained largely unperturbed (**Figure 4E**). Thus, in agreement with the partial development block, ΔE1ΔE3 causes reduced transcription of a subset of Eα targets.

### 3.4 Eα E-box deletion impairs TCR expression in thymic DP cells

**Figure 5:**
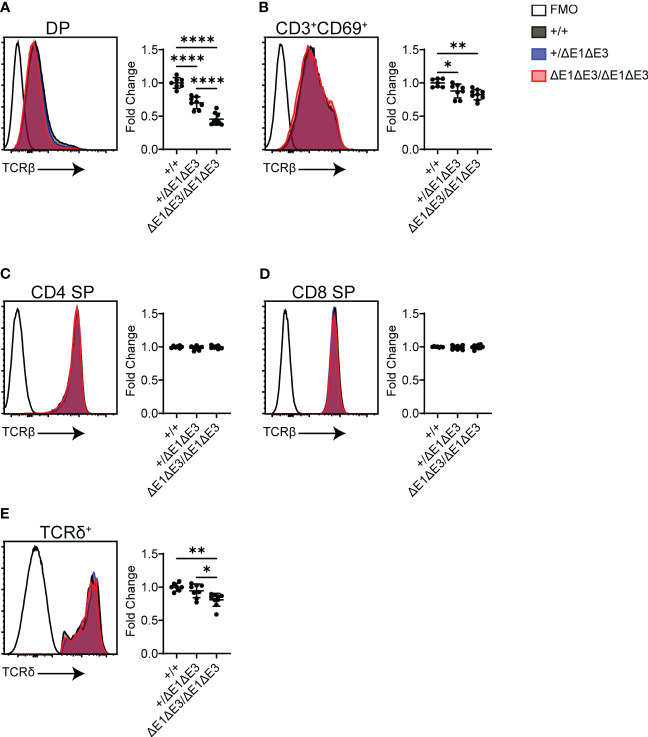
TCR surface expression is reduced in ΔE1ΔE3 DP thymocytes and thymic γδ T lymphocytes. **(A–D)** Representative overlays of TCRβ cell surface staining together with fluorescence-minus-one (FMO) controls, assayed by flow cytometry as shown in **Supplementary Figure 1**. Graphs summarize TCRβ median-MFI for Eα^+/+^, Eα^+/ΔE1ΔE3^, and Eα^ΔE1ΔE3/ΔE1ΔE3^. **(A)** DP thymocytes; **(B)** positively selected (CD3^+^CD69^+^) DP thymocytes; **(C)** CD4^+^ SP thymocytes; **(D)** CD8^+^ SP thymocytes. **(E)** Representative overlay of TCRδ surface staining together with FMO control, with graph summarizing TCRδ median-MFI of TCRδ^+^ thymocytes for Eα^+/+^, Eα^+/ΔE1ΔE3^, and Eα^ΔE1ΔE3/ΔE1ΔE3^. Data pooled from 4 independent experiments. Eα^+/+^ (n = 7), Eα^+/ΔE1ΔE3^ (n = 8), and Eα^ΔE1ΔE3/ΔE1ΔE3^ (n = 9). In all cases, summary graph data for all genotypes are presented with normalization to the average value for Eα^+/+^ (set to 1) within individual experiments. Data are plotted as mean ± SD. Statistical analysis: one-way ANOVA with correction for multiple comparison using Tukey’s *post hoc* testing. *p < 0.05, **p < 0.01, ****p < 0.0001.

Flow cytometry was also used to analyze surface TCR expression in recombinase-sufficient thymocytes. Notably, DP thymocytes of ΔE1ΔE3 mice displayed a substantial reduction in TCRβ surface expression (**Figure 5A**), with a much smaller reduction apparent on CD3^+^CD69^+^ DP thymocytes (**Figure 5B**). There was no difference in TCRβ surface expression on CD4^+^ and CD8^+^ SP thymocytes (**Figures 5C, D**). Because TCR expression is upregulated during positive selection, the reduced frequency of CD3^+^CD69^+^ DP thymocytes (**Figures 2F, G**) could account in part for the overall reduction in TCRβ expression in DP thymocytes. Alternatively, or in addition, the reduction in TCRβ surface expression could reflect an increase of pre-TCR expression in ΔE1ΔE3 DP thymocytes due to impaired *Tcra* gene rearrangement and TCRαβ surface assembly, or diminished TCRαβ expression due to reduced transcription of rearranged *Tcra* genes. That there is no change in TCRβ surface expression in more mature thymocyte populations may reflect selection for higher TCR expression during positive selection, or diminished effects of E proteins on Eα in mature cells (16, 37).

Eα activity is not restricted to αβ T lymphocytes or their development, having been shown to contribute to normal expression of *Tcrd* in γδ T lymphocytes (6). TCRδ^+^ thymocytes of ΔE1ΔE3 mice displayed a small but significant reduction in TCRδ surface expression (**Figure 5E**). This indicates that E proteins contribute in part to Eα activity in γδ T lymphocytes.

### 3.5 Eα E-box deletion impairs TCRα rearrangement

As noted previously, TEA and Jα49 promoter-driven transcription normally target primary rearrangements to Jα segments proximal to these promoters. Once these promoters are deleted by primary rearrangement, secondary rearrangement is thought to be directed by the promoter of the rearranged Vα gene segment. However, in mice with genetic deletion of the TEA (or TEA and Jα49) promoter(s), the activation of cryptic downstream promoters causes dysregulated primary rearrangement directed more broadly across the central and distal Jα segments. As such, changes in Jα transcriptional activity are reflected by changes to the TCRα repertoire (12, 13, 15, 35, 36).

**Figure 6:**
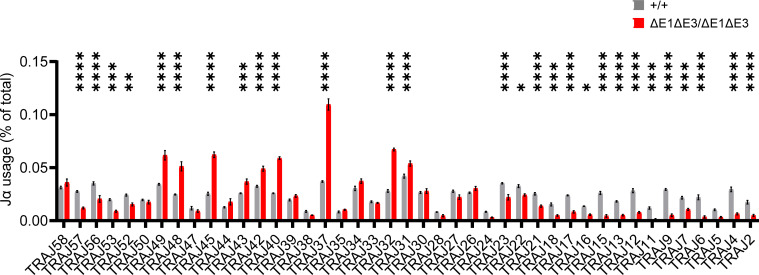
The Jα repertoire is dysregulated in ΔE1ΔE3 mice. Jα segment usage in pre-selection DP thymocytes from Eα^+/+^ (n = 3) and Eα^ΔE1ΔE3/ΔE1ΔE3^ (n = 3) assessed by 5’ RACE. Values shown represent the frequencies with which individual J segments appear in the *Tcra* repertoire and are presented as mean ± SEM. Statistical analysis: two-way ANOVA with correction for multiple comparison using Šidák *post hoc* testing. *p < 0.05, **p < 0.01, ***p < 0.001, ****p < 0.0001.

To assess how E protein binding at Eα impacts TCRα rearrangement, Jα segment usage was evaluated by performing 5′ rapid amplification of cDNA ends (5′RACE) on preselection DP thymocytes (CD4^+^CD8^+^CD3^lo^) sorted from Eα^ΔE1ΔE3/ΔE1ΔE3^ and Eα^+/+^ littermates. The double E-box mutants showed a significantly altered Jα repertoire. While proximal and distal Jα segments were underrepresented, most segments from Jα49 to Jα31 were significantly overrepresented (**Figure 6**, **Supplementary Figure 6**). This suggests a modest defect in primary rearrangements that preferentially affects TEA-dependent Jα segments, coupled with a substantial defect in secondary rearrangements required for usage of distal Jα segments.

### 3.6 iNKT cells are reduced in double Eα E-box mutant

DP thymocytes of *Rorc^-/-^* mice are short-lived and the TCRα repertoire is consequently limited to primary Vα-Jα rearrangements (38). Invariant natural killer T cells (iNKT) are absent in *Rorc^-/-^* mice and thus their characteristic Vα14-Jα18 TCRα chain is considered to be the product of secondary TCRα recombination (39, 40). iNKT cellularity and development was therefore assessed to provide additional evidence that Eα E-box mutations impact secondary Vα-Jα rearrangement.

**Figure 7:**
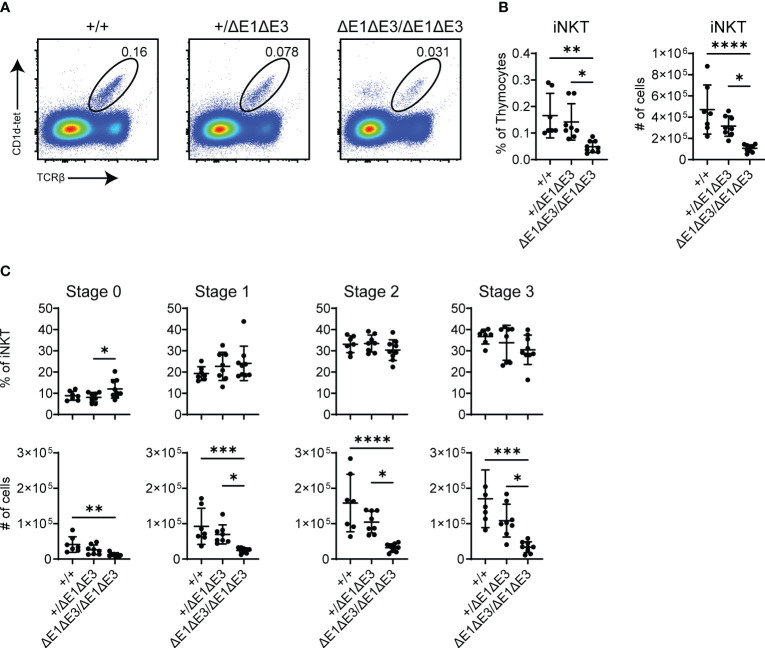
Fewer iNKT cells develop in ΔE1ΔE3 mice. **(A)** Representative flow cytometry plots, displayed as CD1d-tetramer versus TCRβ, of live thymocytes from Eα^+/+^, Eα^+/ΔE1ΔE3^, and Eα^ΔE1ΔE3/ΔE1ΔE3^ mice. Frequencies of gated populations are shown. **(B)** Frequencies and numbers of thymic iNKT cells in Eα^+/+^ (n = 15), Eα^+/ΔE1ΔE3^ (n = 19), and Eα^ΔE1ΔE3/ΔE1ΔE3^ (n = 15) mice, with gating as shown in **(A)**. Data pooled from 8 independent experiments. **(C)** Frequencies and numbers of thymic iNKT stage 0 (CD24^+^), stage 1 (CD24^-^CD44^-^NK1.1^-^), stage 2 (CD24^-^CD44^+^NK1.1^-^), and stage 3 (CD24^-^CD44^+^NK1.1^+^) cells in Eα^+/+^ (n = 7) Eα^+/ΔE1ΔE3^ (n = 8) and Eα^ΔE1ΔE3/ΔE1ΔE3^ (n = 9) mice. Data were pooled from 4 independent experiments and are presented as mean ± SD. Statistical analysis: one-way ANOVA with correction for multiple comparison using Tukey’s *post hoc* testing. *p < 0.05, **p < 0.01, ***p < 0.001, ****p < 0.0001.

While there was no observed change in Eα^ΔE1/ΔE1^ (**Supplementary Figures 7A, B**), flow cytometry analysis determined iNKTs to be significantly reduced in both frequency and number in double E-box mutants (both Eα^ΔE1ΔE3/ΔE1ΔE3^ and Eα^ΔE1ΔE3(1)/ΔE1ΔE3(1)^) (**Figures 7A, B**, **Supplementary Figure 7C**). Because all iNKT developmental stages were reduced numerically and proportionally (**Figure 7C**, **Supplementary Figure 7D**), iNKT development is obstructed prior to TCR surface expression and subsequent lineage commitment. This suggests diminished rearrangement of the Vα14-Jα18 invariant *Tcra* chain in double E-box mutants, and is in accord with reduced usage of Jα18 observed in repertoire analysis. In conjunction with the broadly reduced use of distal Jα segments, this result indicates an impairment of secondary TCRα recombination upon Eα E-box deletion.

Prior work demonstrated a deficiency in iNKT cells in mice lacking E protein HEB (26). Moreover, mice deficient in E protein inhibitors (Id2-Id3 double deficiency) were shown to have an increase in iNKT cells as well as elevated Vα14-Jα18 rearrangement in preselection DP thymocytes (41). However, in neither case was a mechanism clearly established. The present work makes clear that one mechanism by which E proteins control iNKT cell development is by direct effects on the *Tcra* locus.

## 4 Discussion

TCRα rearrangement is a highly regulated and ordered process that takes place during the DP stage of thymocyte development (5). Eα is a required *cis*-acting element which plays crucial roles in promoting locus accessibility and establishing the RC (14, 42). Among the TFs binding at Eα, E proteins have three identified binding sites and show recruitment as early as the DN stage (11, 16, 27). E2A and HEB carry out a myriad of roles crucial to lymphocyte development, including mediating germline transcription and recombination accessibility at other antigen receptor loci (17, 22, 43). The findings shown here indicate that Eα E protein binding contributes to the regulation of TCRα recombination and repertoire diversity.

Although there was no effect of single E-box deletion (ΔE1), double E-box disruption [ΔE1ΔE3 and ΔE1ΔE3(1)] produced a partial block in αβ T cell development at the DP stage, and thus impaired generation of SP thymocytes. The present data cannot distinguish whether the effects of E protein binding to E1 and E3 are distinct or redundant. Generation of ΔE3 and ΔE2 single mutants, as well as combinatorial E-box deletions, would be needed to more fully understand the contributions of the three E protein binding sites. We did not disrupt E2 in the present study because mutation of two E-protein binding sites proved sufficient to test the central hypothesis of the study, namely that E protein binding to Eα is important for Eα activity and *Tcra* recombination. The finding that Eα GATA3 binding is at least partially dependent on E protein binding suggests that one function of E proteins is to stabilize the binding of other transcription factors on Eα, and that reduced binding of other transcription factors may contribute to impaired Eα activity when E protein binding is prevented.

Intriguingly, germline transcription in ΔE1ΔE3 was primarily reduced across the Jα array, even though Eα is known to regulate chromatin structure and transcription over hundreds of kb upstream and downstream. Interaction with and accessibility at these other regions may be regulated or compensated by other Eα binding TFs. Furthermore, while transcript abundance is reduced at both TEA and Jα49, the overrepresentation of central Jα segments in the TCRα repertoire indicates that recombination is being preferentially directed by the latter promoter (12). Consistent with this, Jα49 is the most proximal Jα segment to be present at a higher proportion in the repertoire. Collectively, these data suggest that E proteins binding at Eα has a prominent role in regulating TEA functionality and thus the accessibility of TEA-dependent Jα segments.

The TEA and Jα49 promoters are crucial to locus accessibility and directing primary recombination to proximal Jα elements. In the absence of the TEA promoter, or the TEA and Jα49 promoters, primary recombination is dysregulated and there is elevated usage of Jα gene segments across the central and distal portions of the Jα array (12, 15). However, relative usage of Jα segments distal to Jα31 was reduced in ΔE1ΔE3 mice. This, together with the reduction in iNKT cells, indicates a substantial impairment of secondary Vα-Jα recombination in ΔE1ΔE3 mice. This suggests that E protein binding at Eα may additionally regulate the promoters of rearranged Vα gene segments, even though there is no effect on those promoters when they are more distant in the unrearranged locus.

The reported findings show that E protein binding at Eα is important to but not necessary for αβ T cell development. Ablation of all Eα E-boxes may produce a more prominent developmental block at DP, but this may not be equivalent to ΔEα given that other TFs may retain binding ability and thus maintain sufficient Eα function to permit TCRα chain recombination, albeit at reduced levels. The importance of E protein binding is further emphasized by the broad conservation of E-boxes at Eα in mammals (**Supplementary Figure 8**). The present results add to the literature on E protein control of AgR gene recombination, with E proteins now shown to regulate all AgR loci. In contributing to locus accessibility and regulation of Vα-Jα rearrangement, Eα-bound E proteins increase TCRα repertoire diversity and the potential for antigen recognition by αβ T cells.

## Data availability statement

The datasets presented in this study can be found in online repositories. The names of the repository/repositories and accession number(s) can be found below: https://www.ncbi.nlm.nih.gov/geo/, GSE227164.

## Ethics statement

The animal study was reviewed and approved by Duke University Institutional Animal Care and Use Committee.

## Author contributions

AM, SR, MK, and YZ contributed to conception and design of the study. Investigation and analysis was carried out by AM. AM and MK contributed to writing of manuscript original draft. AM, SR, MK, and YZ contributed to review, editing, and approval of submitted manuscript.
