# Supplementary material for: E protein binding at the Tcra enhancer promotes Tcra repertoire diversity
Source: Front Immunol. 2023 Jul 6;14:1188738. doi: 10.3389/fimmu.2023.1188738 (PMC10358851; doi:10.3389/fimmu.2023.1188738)
Supplement: Supplementary file 1 [file DataSheet_1.docx]

Supplementary Material

E protein binding at the *Tcra* enhancer promotes *Tcra* repertoire diversity

Ariana Mihai, Sumedha Roy, Michael S Krangel*, Yuan Zhuang

*** Correspondence:** Michael S. Krangel: krang001@mc.duke.edu

# Supplementary Figures


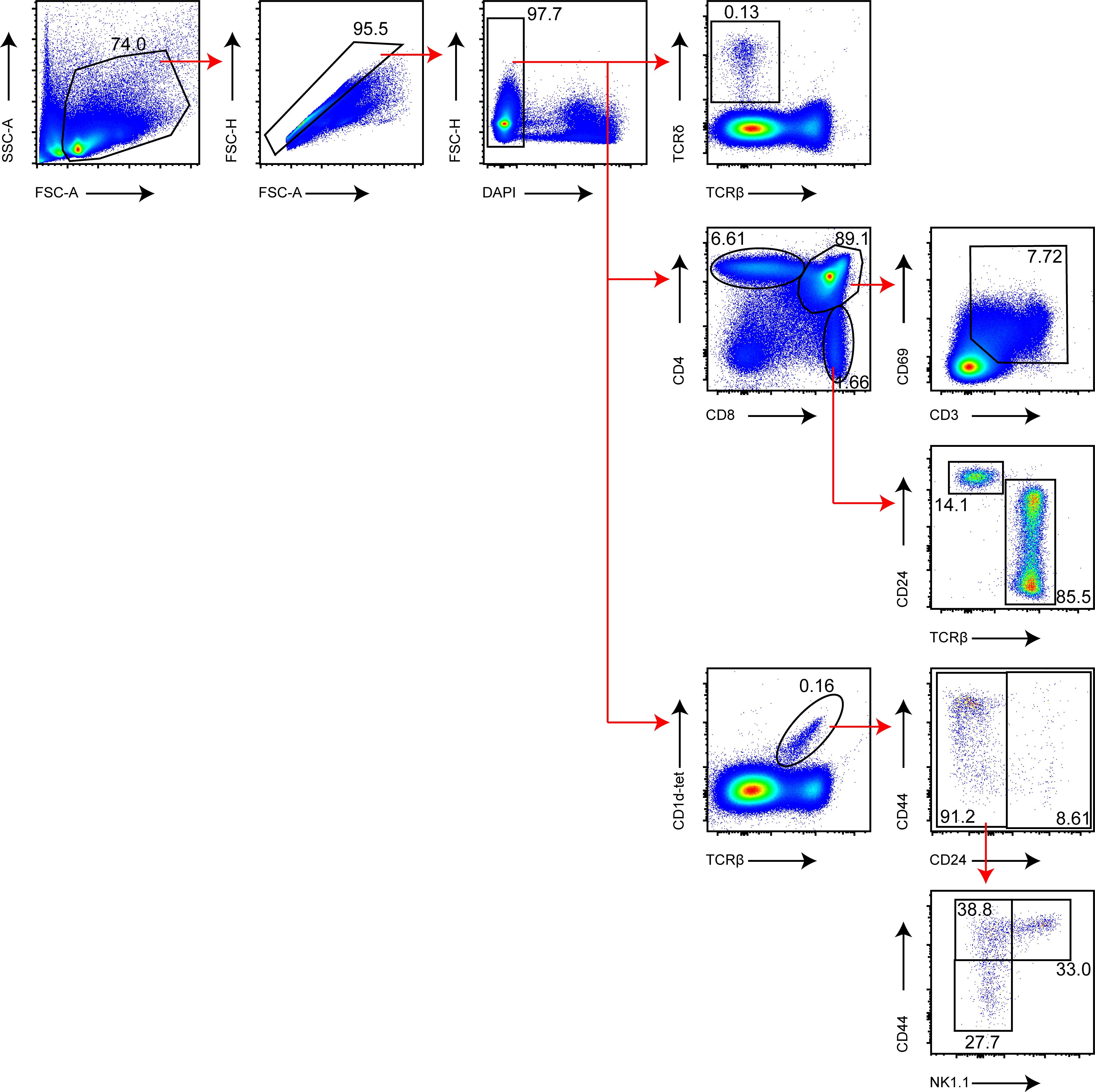


**Supplementary Figure 1.** Gating scheme used for flow cytometry analysis. Debris, doublets, and dead cells were excluded by SSC-A vs FSC-A, FSC-H vs FSC-A, and FSC-H vs live/dead marker, respectively. γδ T cells were gated as TCRδ^+^ (TCRδ vs TCRβ) of live thymocytes. CD4^+^ SP and DP cells were gated as CD4^+^ and CD4^+^CD8^+^ (CD4 vs CD8) of live thymocytes. CD8^+^ SP cells were gated as CD24^-^TCRβ^+^ (CD24 vs TCRβ) from CD8^+^ live thymocytes. Positively selected DP cells were gated as CD3^+^CD69^+^ of DP cells (CD69 vs CD3). iNKT cells were gated as CD1d-tet^+^TCRβ^+^ (CD1d-tet vs TCRβ) of live thymocytes. Stage 0 iNKT cells were gated as CD24^+^ of iNKT cells (CD44 vs CD24). Stage 1, 2, and 3 iNKT cells were gated as CD44^-^NK1.1^-^, CD44^+^NK1.1^-^, and CD44^+^NK1.1^+^, respectively, of CD24^-^ iNKT cells. Gating is related to Figures 1, 2, 4, 6, and Supplementary Figure 3.


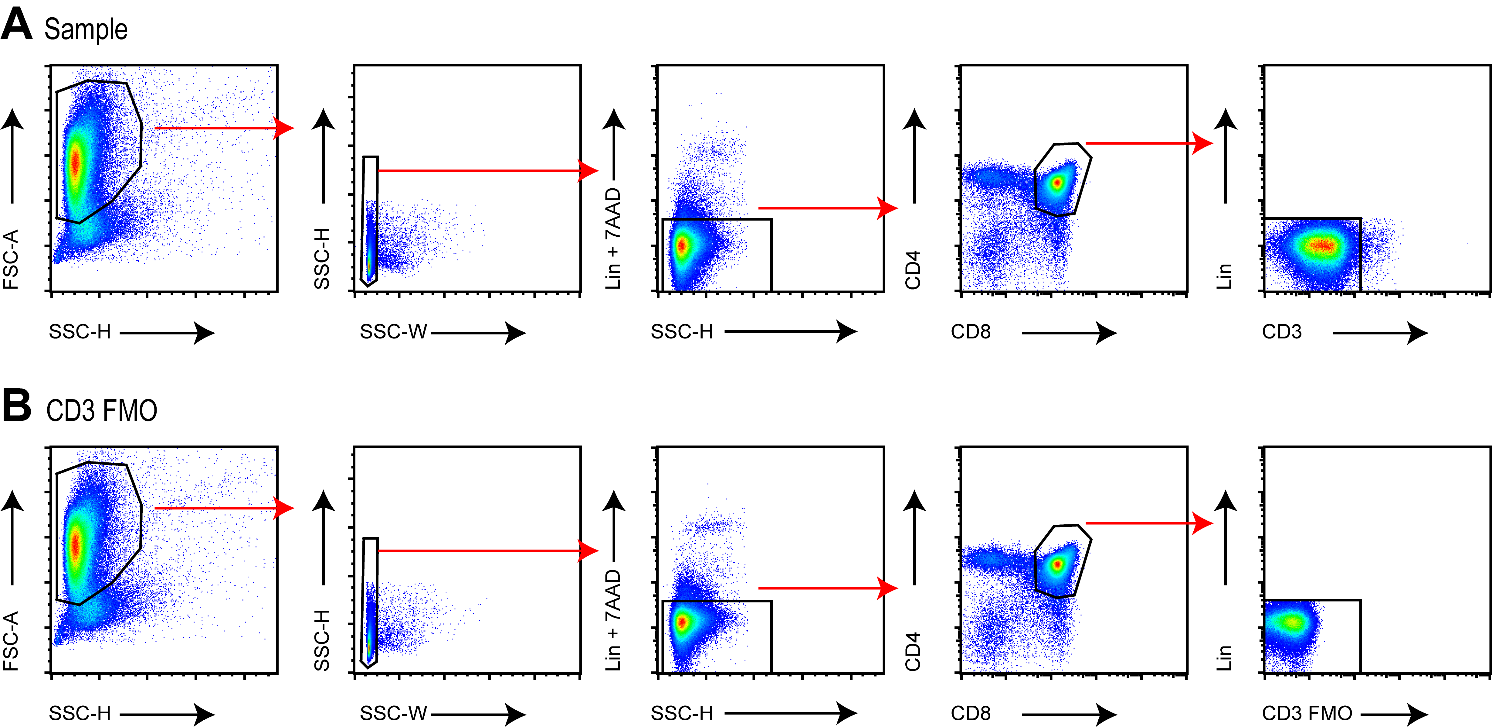


**Supplementary Figure 2.** Gating strategy for sorting of CD3^lo^ DP thymocytes. Exclusion of debris (FSC-A vs SSC-H), doublets (SSC-H vs SSC-W), and Lin^+^ (described in Materials and Methods) and/or dead cells (Lin + 7AAD vs SSC-H). DP cells were gated as CD4^+^CD8^+^ (CD4 vs CD8) of live Lin^-^ thymocytes. DP cells were then gated as CD3^lo^ (Lin/live marker vs CD3). Fully stained sample (A) and CD3 fluorescence minus one (FMO) (B) shown. Gating is related to Figure 6 and Supplementary Figure 6 for sorting of CD3^lo^ DP thymocytes.


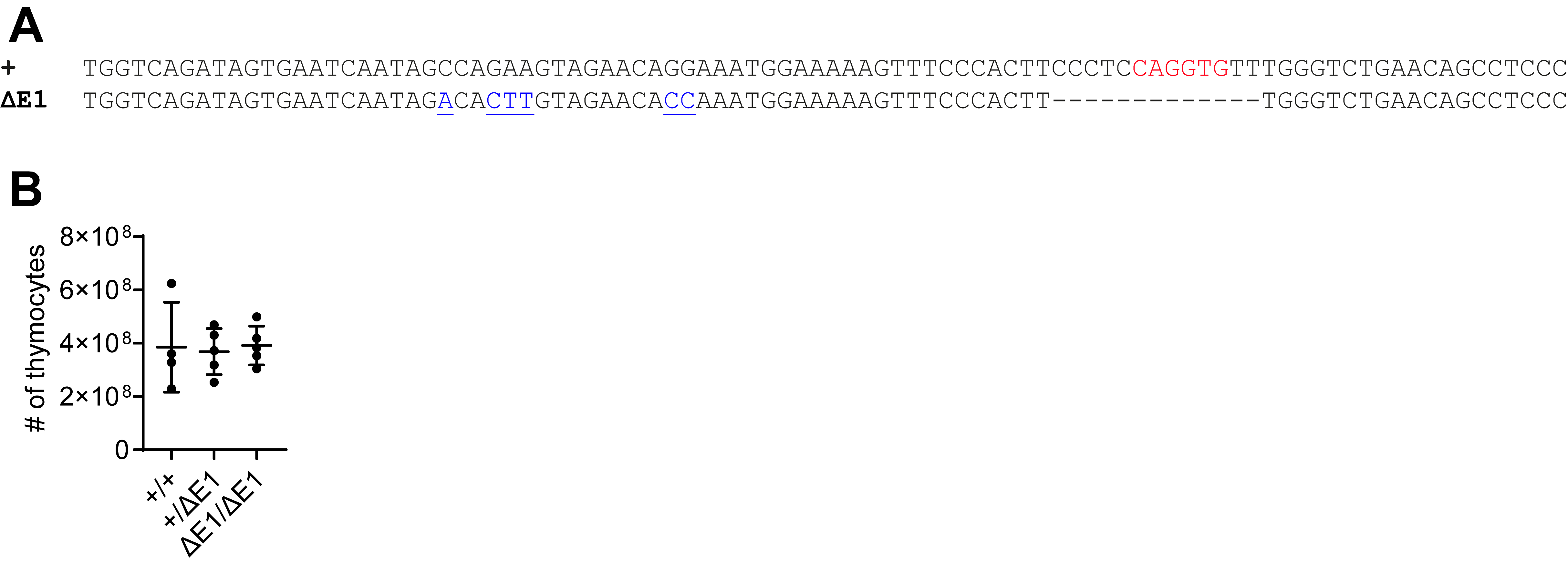


**Supplementary Figure 3.** ΔE1 mutant sequence and thymus cellularity. (A) Extended sequence comparison of wild-type (+) and ΔE1 mutant. E protein binding motif is highlighted in red, and base pair changes occurring at the gRNA recognition sequence are underlined and highlighted in blue. (B) Total thymic cellularity in Eα^+/+^ (n = 4), Eα^+/ΔE1^ (n = 5), and Eα^ΔE1/ΔE1^ (n = 5) mice. Data were pooled from 3 independent experiments and presented as mean±SD. Statistical analysis: One-way ANOVA with correction for multiple comparisons using Tukey’s post hoc testing. Significant differences were not detected.


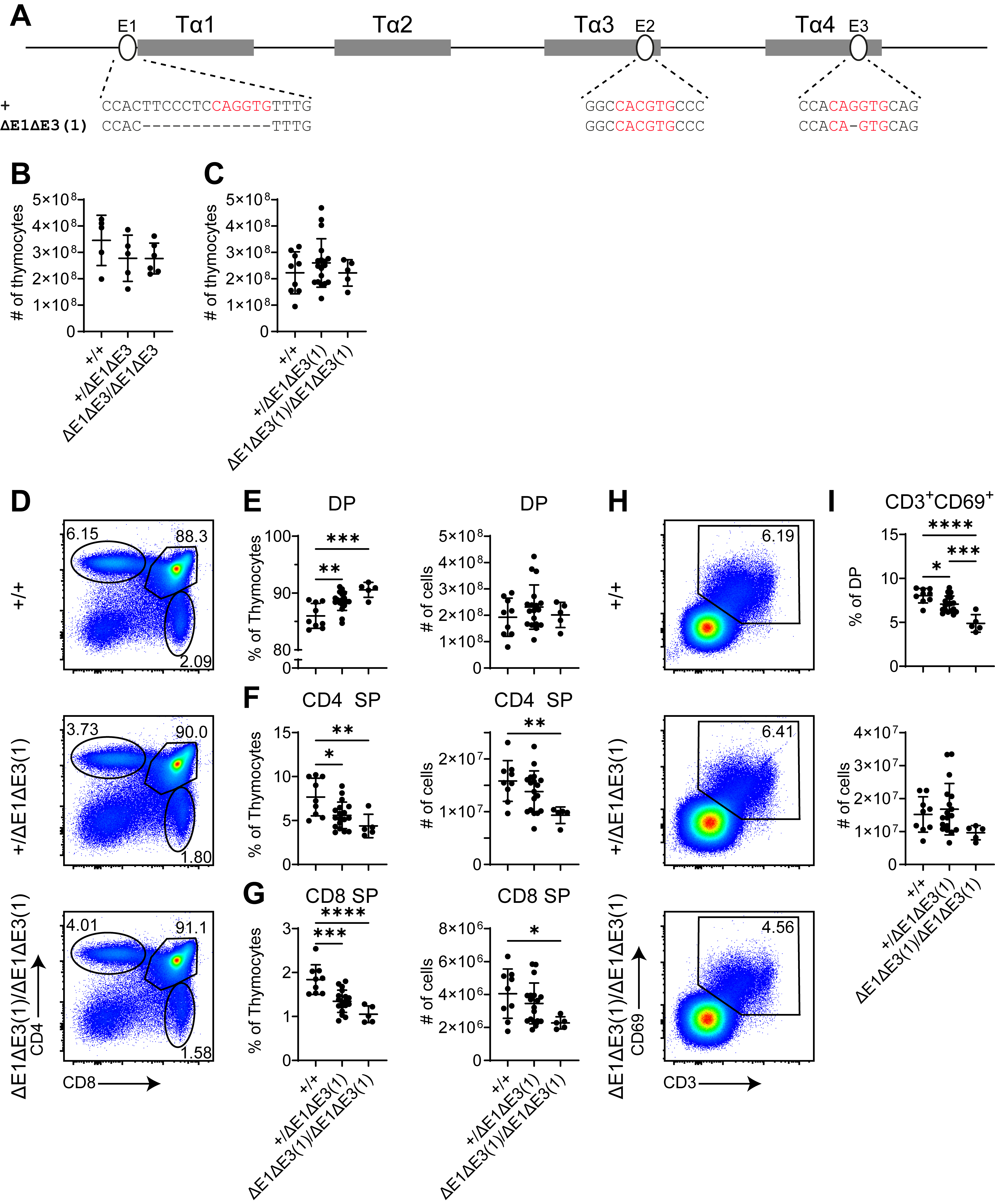


**Supplementary Figure 4.** T cell development in ΔE1ΔE3(1) mutant. (A) Diagram of relative positions of Tα1-Tα4 protein binding regions and E1-E3 E protein binding sites within Eα, with sequences of wild-type (+) and ΔE1ΔE3(1) mutation indicated below. E protein binding motifs are highlighted in red. (B) Total thymic cellularity in Eα^+/+^ (n = 5), Eα^+/ΔE1ΔE3^ (n = 5), and Eα^ΔE1ΔE3/ΔE1ΔE3^ (n = 6). Data were pooled from 3 independent experiments. (C) Total thymic cellularity in Eα^+/+^, Eα^+/ΔE1ΔE3(1)^, and Eα^ΔE1ΔE3(1)/ΔE1ΔE3(1)^. (D) Representative flow cytometry plots, displayed as CD4 versus CD8, of live thymocytes from Eα^+/+^, Eα^+/ΔE1ΔE3(1)^, and Eα^ΔE1ΔE3(1)/ΔE1ΔE3(1)^ mice. Frequencies of gated populations are shown. (E-G) Frequencies and numbers of (E) DP thymocytes, (F) CD4^+^ SP thymocytes, and (G) CD8^+^ SP thymocytes in Eα^+/+^, Eα^+/ΔE1ΔE3(1)^, and Eα^ΔE1ΔE3(1)/ΔE1ΔE3(1)^. (H) Representative flow cytometry plots, displayed as CD69 versus CD3, of DP thymocytes (gated as shown in (D)) from Eα^+/+^, Eα^+/ΔE1ΔE3(1)^, and Eα^ΔE1ΔE3(1)/ΔE1ΔE3(1)^ mice. Frequencies of gated populations are shown. (I) Frequencies and numbers of CD3^+^CD69^+^ DP cells in Eα^+/+^, Eα^+/ΔE1ΔE3(1)^, and Eα^ΔE1ΔE3(1)/ΔE1ΔE3(1)^, with gating as shown in (H). (C-I) Data were pooled from 3 independent experiments and are plotted as mean±SD. Eα^+/+^ (n = 9), Eα^+/ΔE1ΔE3(1)^ (n = 18), Eα^ΔE1ΔE3(1)/ΔE1ΔE3(1)^ (n = 5). Statistical analysis: one-way ANOVA with correction for multiple comparison using Tukey’s post hoc testing. **p* < 0.05, ***p* < 0.01, ****p* < 0.001, *****p* < 0.0001.

**
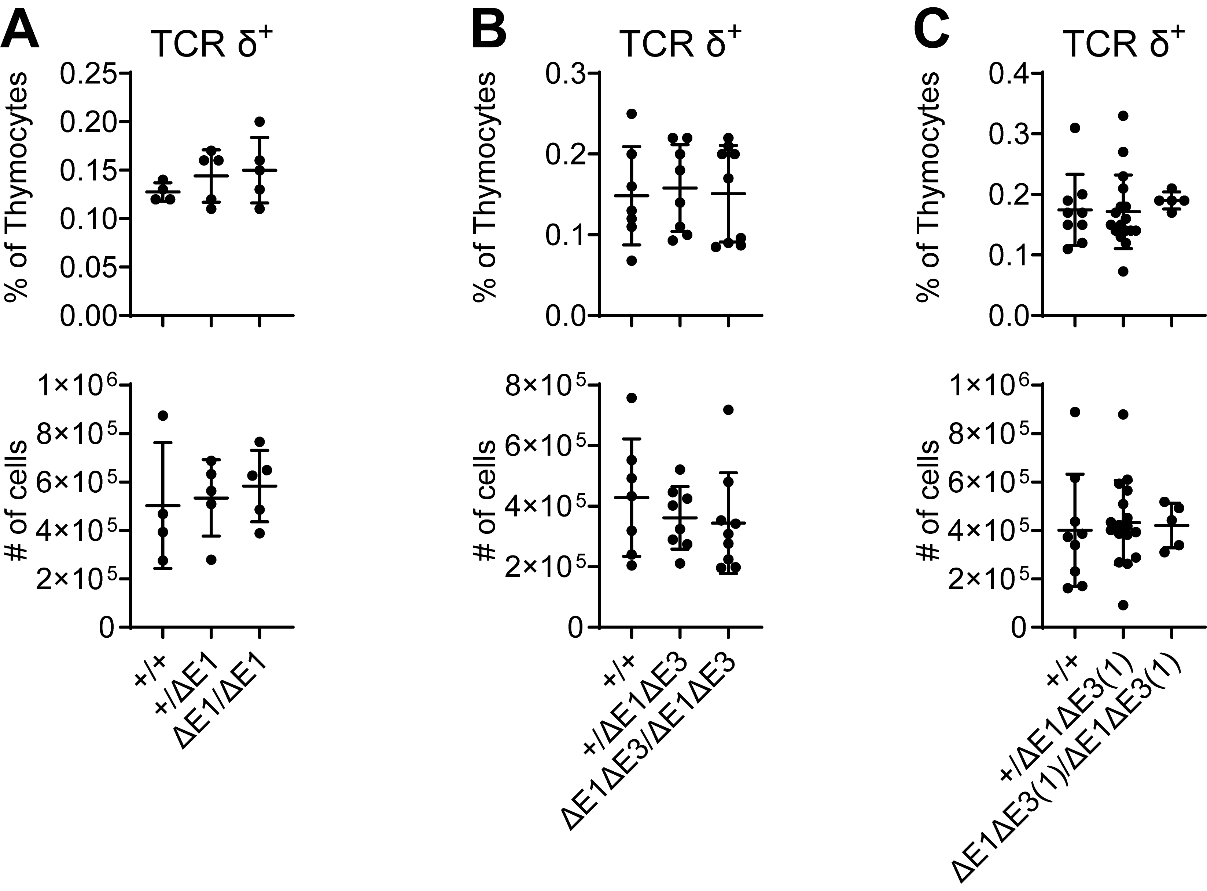
**

**Supplementary Figure 5.** γδ T cell development in Eα E-box mutants. Frequency and number of γδ T lymphocytes in (A) Eα^+/+^ (n = 4), Eα^+/ΔE1^ (n = 5), and Eα^ΔE1/ΔE1^ (n = 5) from 3 independent experiments; (B) Eα^+/+^ (n = 15), Eα^+/ΔE1ΔE3^ (n = 19), and Eα^ΔE1ΔE3/ΔE1ΔE3^ (n = 15) from 8 independent experiments; (C) in Eα^+/+^ (n = 9), Eα^+/ΔE1ΔE3(1)^ (n = 17), and Eα^ΔE1ΔE3(1)/ΔE1ΔE3(1)^ (n = 5) from 3 independent experiments. Data are presented as mean±SD. Statistical analysis: one-way ANOVA with correction for multiple comparisons using Tukey’s post hoc testing. Significant differences were not detected.


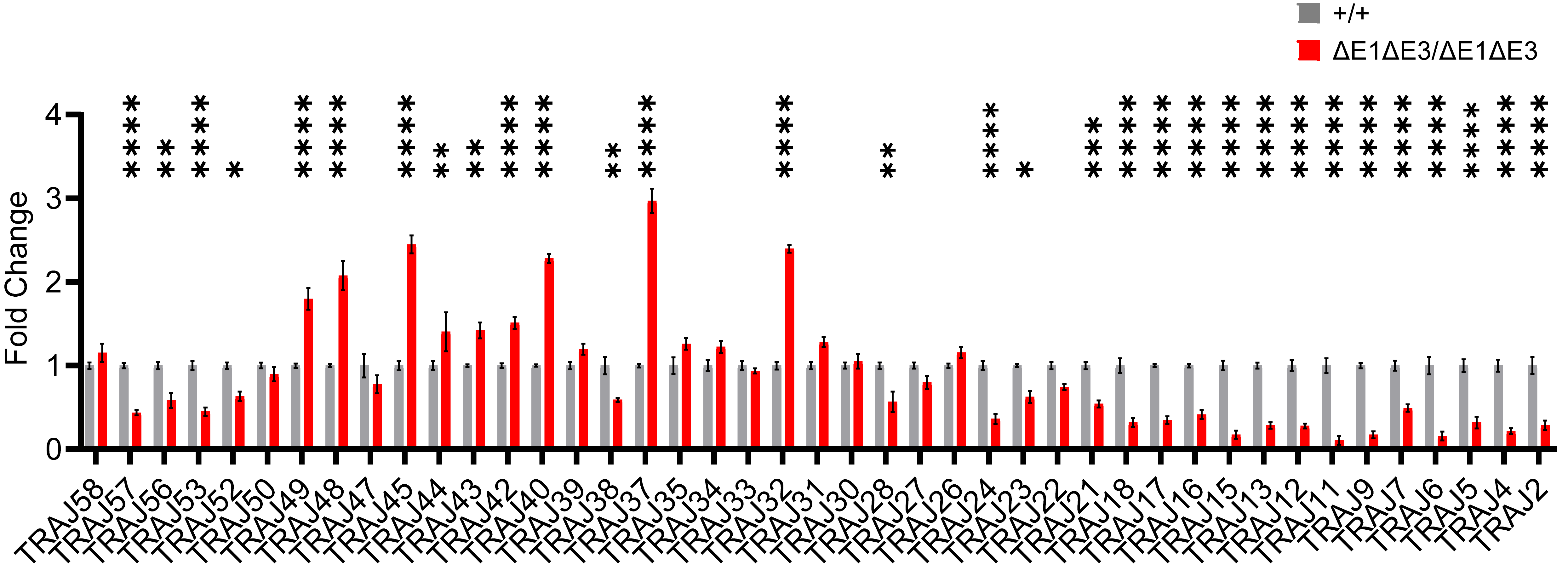


**Supplementary Figure 6.** Jα repertoire in ΔE1ΔE3 mice. Data for Jα segment usage in pre-selection DP cells from Eα^+/+^ (n = 3) and Eα^ΔE1ΔE3/ΔE1ΔE3^ (n = 3) in Figure 5 are replotted with normalization to the average value for Eα^+/+^ for each Jα segment (set to 1). Data are presented as mean±SEM. Statistical analysis: two-way ANOVA with correction for multiple comparison using Šidák post hoc testing. **p* < 0.05, ***p* < 0.01, ****p* < 0.001, *****p* < 0.0001.


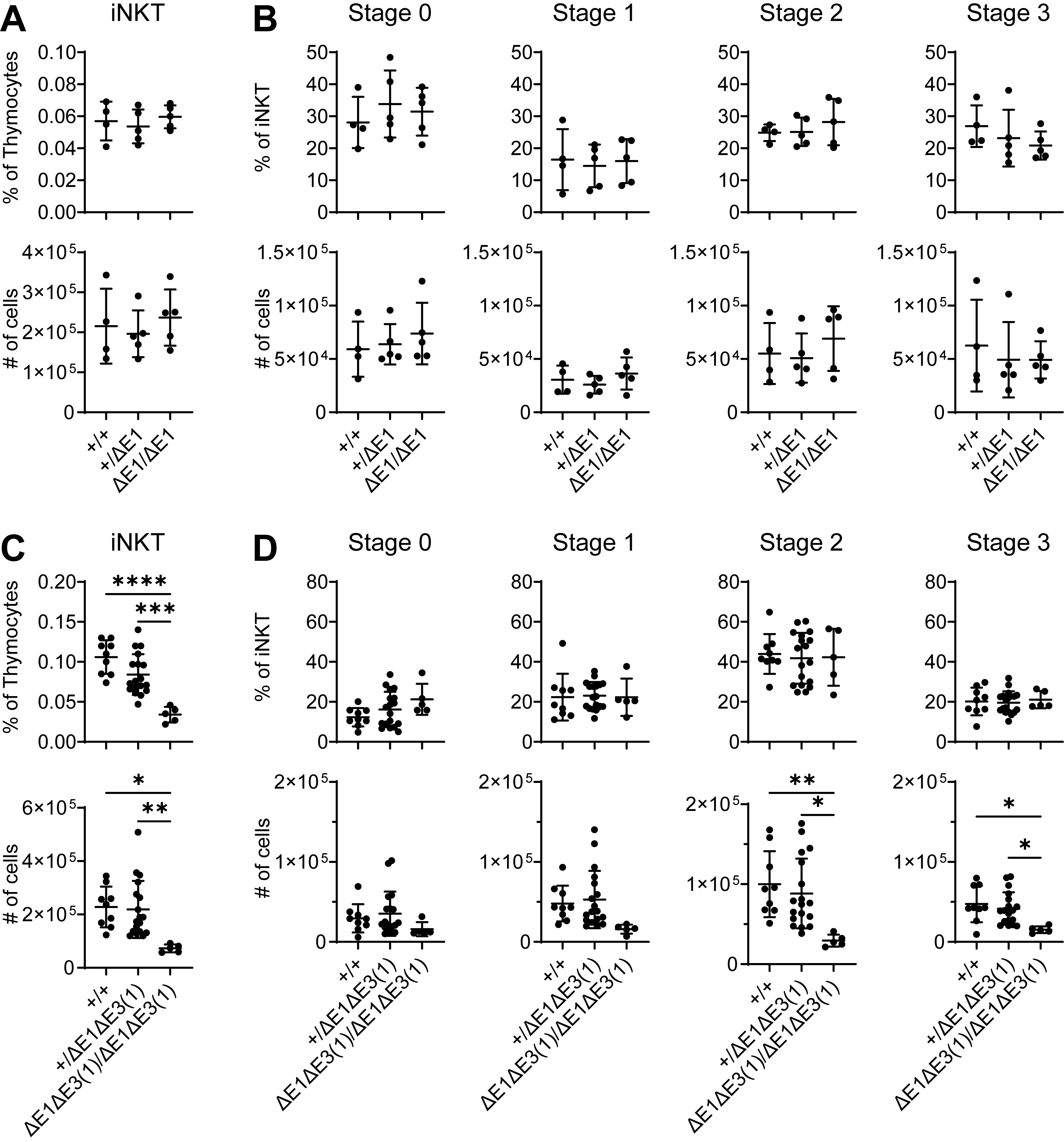


**Supplementary Figure 7.** iNKT cell development in ΔE1 and ΔE1ΔE3(1) mutants. Frequencies and numbers of (A) thymic iNKT cells, and (B) thymic iNKT stage 0 (CD24^+^), stage 1 (CD24^-^CD44^-^NK1.1^-^), stage 2 (CD24^-^CD44^+^NK1.1^-^), and stage 3 (CD24^-^CD44^+^NK1.1^+^) cells, in Eα^+/+^, Eα^+/ΔE1^, and Eα^ΔE1/ΔE1^ mice. Data were pooled from 3 independent experiments. Eα^+/+^ (n = 4), Eα^+/ΔE1^ (n = 5), and Eα^ΔE1/ΔE1^ (n = 5). Frequencies and numbers of (C) thymic iNKT cells and (D) thymic iNKT stage 0 (CD24^+^), stage 1 (CD24^-^CD44^-^NK1.1^-^), stage 2 (CD24^-^CD44^+^NK1.1^-^), and stage 3 (CD24^-^CD44^+^NK1.1^+^) cells in Eα^+/+^, Eα^+/ΔE1ΔE3(1)^, and Eα^ΔE1ΔE3(1)/ΔE1ΔE3(1)^ mice. Data were pooled from 3 independent experiments. Eα^+/+^ (n = 9), Eα^+/ΔE1ΔE3(1)^ (n = 18), and Eα^ΔE1ΔE3(1)/ΔE1ΔE3(1)^ (n = 5). Data are presented as mean±SD. Statistical analysis: one-way ANOVA with correction for multiple comparison using Tukey’s post hoc testing. **p* < 0.05, ***p* < 0.01, ****p* < 0.001, *****p* < 0.0001.


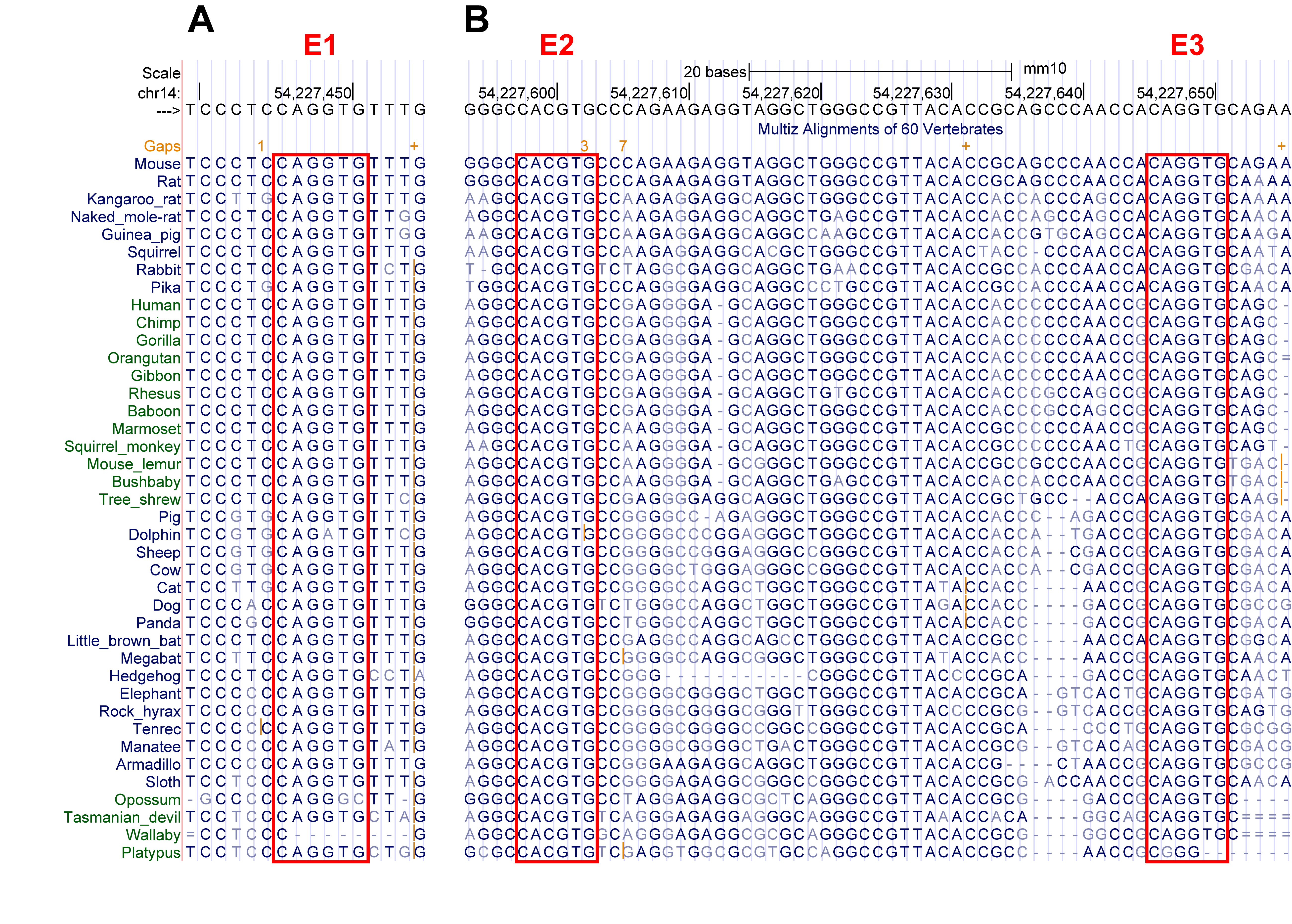


**Supplementary Figure 8.** Comparative analysis of E1, E2, and E3 among vertebrates. (A) UCSC genome browser view showing sequence conservation of E1 (sequence framed in red). (B) UCSC genome browser view showing sequence conservation of E2 and E3 (sequence framed in red).
