# Supplementary material for: E protein binding at the Tcra enhancer promotes Tcra repertoire diversity
Source: Front Immunol. 2023 Jul 6;14:1188738. doi: 10.3389/fimmu.2023.1188738 (PMC10358851; doi:10.3389/fimmu.2023.1188738)
Supplement: Supplementary file 2 [file DataSheet_2.docx]

Supplementary Material

E protein binding at the *Tcra* enhancer promotes *Tcra* repertoire diversity

Ariana Mihai, Sumedha Roy, Michael S Krangel*, Yuan Zhuang

*** Correspondence:** Michael S. Krangel: krang001@mc.duke.edu

# Supplementary Tables

**Supplementary Table 1.** Primer, guide, and donor sequences.

| Name | Sequence | Application | Reference |
| --- | --- | --- | --- |
| Donor E1 | (50 bp homology)-GACCCGCTAGCAAGCTTGGAAGTGGGAAACTTTTTCCATTTGGTGTTCTACAAGTGTC-(50 bp homology) | CRISPR/Cas9 Donor | Present Study |
| gRNA E1 (5') | TCCATTTCCTGTTCTACTTC | CRISPR/Cas9 gRNA (E1) 5' | Present Study |
| gRNA E1 (3') | CCCACTTCCCTCCAGGTGTTT | CRISPR/Cas9 gRNA (E1) 3' | Present Study |
| gRNA E3 | GGCCTTCTTTTCTGCACCTG | CRISPR/Cas9 gRNA (E3) | Present Study |
| Ea Mutant (F) | GGAAAAAGTTTCCCACTTTGGG | Genotype | Present Study |
| Ea Wildtype (F) | AAGTTTCCCACTTCCCTCCAGGTG | Genotype | Present Study |
| Ea (R) | GGGACTTCTTGGGAGTGGAG | Genotype | Present Study |
| rs239422031 (F) | CAGGGGTTTTTCCACTGGACT | C57BL/6 - SJL/J polymorphisms | Present Study |
| rs214457683 (F) | GAAGCGGCTAATAGCGAGCA | C57BL/6 - SJL/J polymorphisms | Present Study |
| rs252954196 (F) | TGGAAGTCACAGTGCTTGGG | C57BL/6 - SJL/J polymorphisms | Present Study |
| rs246092826 (F) | TCACTGTTTAAAACCCGTTGGG | C57BL/6 - SJL/J polymorphisms | Present Study |
| rs239422031 (R) | CCACAACCTGGGTTCCTGTT | C57BL/6 - SJL/J polymorphisms | Present Study |
| rs214457683 (R) | GCATGGGGGACCATAGTCAA | C57BL/6 - SJL/J polymorphisms | Present Study |
| rs252954196 (R) | GTTGAATCCTCCCCAGCCAA | C57BL/6 - SJL/J polymorphisms | Present Study |
| rs246092826 (R) | TGTAATGCCAGCGGGATCAT | C57BL/6 - SJL/J polymorphisms | Present Study |
| Trac (F) | CCTGTCAGTTATGGGACTCCG | RT-qPCR | Present Study |
| Trac (R) | GGGAGTCAGGCTCTGTCAGT | RT-qPCR | Present Study |
| Trav17 (F) | TGGAGCGACTCAGCCAAGTA | RT-qPCR | Previously reported (44) |
| Trav17 (R) | CGTGCACAGAAGGTCTCAGG | RT-qPCR | Previously reported (44) |
| Trav19 (F) | CCAGCCTGAAGACACAGCAG | RT-qPCR | Previously reported (44) |
| Trav19 (R) | AGGGTCGCAGATGTCCTTGT | RT-qPCR | Previously reported (44) |
| Trav21 (F) | CGGCTGTGTACCACTGTATCCT | RT-qPCR | Previously reported (44) |
| Trav21 (R) | CTTGAAATGGATGCCTCTGCT | RT-qPCR | Previously reported (44) |
| Trdj1 (F) | AGCTGCTGAGGTTTTTGGAATG | RT-qPCR | Previously reported (44) |
| Trdj1 (R) | ATCCCTCAGACCCTAACCCAGA | RT-qPCR | Previously reported (44) |
| Trdv5 (F) | CTGGGAACTCTCTTAACCATCCC | RT-qPCR | Previously reported (44) |
| Trdv5 (R) | ACCGACTGGAAGGATGATTCTT | RT-qPCR | Previously reported (44) |
| TEA (F) | GCACCTTCCTTCCAAGATTCCT | RT-qPCR | Previously reported (44) |
| TEA (R) | GTGCTGGTCCTTGTCTTTCGTT | RT-qPCR | Previously reported (44) |
| Traj56 (F) | CCCTTGGAACCCTGATATGC | RT-qPCR | Previously reported (44) |
| Traj56 (R) | CAGCCATTGTTTGGATTGGA | RT-qPCR | Previously reported (44) |
| Traj31 (F) | GCAGCCTGCCAGCTATCTTT | RT-qPCR | Previously reported (44) |
| Traj31 (R) | CAAAAGCAGCAACCCAACAA | RT-qPCR | Previously reported (44) |
| Traj23 (F) | AGAGGAGGCCGAAAGTCTCC | RT-qPCR | Previously reported (44) |
| Traj23 (R) | TGCACAAATCCAGGCCTATG | RT-qPCR | Previously reported (44) |
| Traj3 (F) | TCTGGACCCTTGGCAATCAT | RT-qPCR | Previously reported (44) |
| Traj3 (R) | CCCTGCCCTGGTCTACTGTG | RT-qPCR | Previously reported (44) |
| Actb (F) | ACACCCGCCACCAGTTC | RT-qPCR | Previously reported (44) |
| Actb (R) | TACAGCCCGGGGAGCAT | RT-qPCR | Previously reported (44) |
| Dad1 (F) | TGTGGGCAGCTTCATCCTAG | RT-qPCR | Previously reported (44) |
| Dad1 (R) | GTGCTGGCAAAGAGGAAGTC | RT-qPCR | Previously reported (44) |
| Abdh4 (F) | TGGAAGCCAGGATCCTCCAG | RT-qPCR | Previously reported (44) |
| Abdh4 (R) | CATCACCAGAGGGGTGCGAT | RT-qPCR | Previously reported (44) |
| Prmt5 (F) | GGTGGTTGGTTCCCGTGATG | RT-qPCR | Previously reported (44) |
| Prmt5 (R) | GCCATTCTCCCCACCAGCAT | RT-qPCR | Previously reported (44) |
| Ajuba (F) | TGCTCTGCCCATAGATACCT | RT-qPCR | Previously reported (44) |
| Ajuba (R) | GTCTCCTGGTCCCTTCGTTC | RT-qPCR | Previously reported (44) |
| Cdh24 (F) | CGTCTTCGGGCTCAATGGA | RT-qPCR | Previously reported (44) |
| Cdh24 (R) | GGGCCAGATCTCAGCCAGT | RT-qPCR | Previously reported (44) |
| Acin1 (F) | GATGGAGCTGCAGCCTCCT | RT-qPCR | Previously reported (44) |
| Acin1 (R) | CCCGTTCCGCGTCAAGCAG | RT-qPCR | Previously reported (44) |
| Homez (F) | AGCAGGTGCTCATTTCCATCC | RT-qPCR | Previously reported (44) |
| Homez (R) | AGCAGTCTCAACAGCTCTGCA | RT-qPCR | Previously reported (44) |
| Pabpn1 (F) | TCAAAGCTCGAGTCAGGGAGA | RT-qPCR | Previously reported (44) |
| Pabpn1 (R) | ACGTAGATAGAGCGGGCATCA | RT-qPCR | Previously reported (44) |
| Ngdn (F) | CACTGACGACAAAAGTTCGAGC | RT-qPCR | Previously reported (44) |
| Ngdn (R) | AGAGGCCTTGTCCAGGATGA | RT-qPCR | Previously reported (44) |
| Trac-RT | TTTCGGCACATTGATTTG | Repertoire Sequencing | Previously reported (29) |
| SmartNNNa | AAGCAGUGGTAUCAACGCAGAGUNNNNUNNNNUNNNNUCTTrGrGrG | Repertoire Sequencing | Previously reported (29) |
| Tcra-n1R | ATTGGGCAGCCCTGATTGGTGCTGTCCTGAGACCGAG | Repertoire Sequencing | Previously reported (29) |
| M1SS | AAGCAGTGGTATCAACGCA | Repertoire Sequencing | Previously reported (30) |
| M1S-B1 | NNNNTTGACTCAGTGGTATCAACGCAG | Repertoire Sequencing | Adapted from (29, 30, 45) |
| M1S-B2 | NNNNGGAACTCAGTGGTATCAACGCAG | Repertoire Sequencing | Adapted from (29, 30, 45) |
| M1S-B3 | NNNNTGACATCAGTGGTATCAACGCAG | Repertoire Sequencing | Adapted from (29, 30, 45) |
| M1S-B4 | NNNNGGACGGCAGTGGTATCAACGCAG | Repertoire Sequencing | Adapted from (29, 30, 45) |
| M1S-B5 | NNNNGCGGACCAGTGGTATCAACGCAG | Repertoire Sequencing | Adapted from (29, 30, 45) |
| M1S-B6 | NNNNTTTCACCAGTGGTATCAACGCAG | Repertoire Sequencing | Adapted from (29, 30, 45) |
| MZ-B4 | NNNNCCACTCATTGGGCAGCCCTGATT | Repertoire Sequencing | Adapted from (29, 30, 45) |
| MZ-B5 | NNNNATCAGTATTGGGCAGCCCTGATT | Repertoire Sequencing | Adapted from (29, 30, 45) |
| MZ-B6 | NNNNAGGAATATTGGGCAGCCCTGATT | Repertoire Sequencing | Adapted from (29, 30, 45) |
| MZ-B7 | NNNNCTTTTGATTGGGCAGCCCTGATT | Repertoire Sequencing | Adapted from (29, 30, 45) |
| MZ-B8 | NNNNNTAGTTGATTGGGCAGCCCTGATT | Repertoire Sequencing | Adapted from (29, 30, 45) |
| MZ-B9 | NNNNNNATCGTGATTGGGCAGCCCTGATT | Repertoire Sequencing | Adapted from (29, 30, 45) |
| Ta1 (F) | ATGGGTAAGCTGGTCAGATAGTGAA | ChIP-qPCR | Present Study |
| Ta1 (R) | GGAAGTGGGAGGCTGTTCAGA | ChIP-qPCR | Present Study |
| Ta3 (F) | CAGAAGCCACATCCTCTGGAAAG | ChIP-qPCR | Present Study |
| Ta3 (R) | CGGCCCAGCCTACCTCTTC | ChIP-qPCR | Present Study |
| Ta4 (F) | GTGCCCAGAAGAGGTAGGCTG | ChIP-qPCR | Present Study |
| Ta4 (R) | CCAAGACCTGCAAGCCCCAC | ChIP-qPCR | Present Study |
| ASE (F) | CCACCTGTGTTAACCGTCAG | ChIP-qPCR | Previously reported (46) |
| ASE (R) | CTATCTTTGCAGCCCACCAA | ChIP-qPCR | Previously reported (46) |
| Gapdh (F) | TGGCGTAGCAATCTCCTTTT | ChIP-qPCR | Previously reported (47) |
| Gapdh (R) | CTCCTGGCTTCTGTCTTTGG | ChIP-qPCR | Previously reported (47) |

**References**

44. Zhao H, Li Z, Zhu Y, Bian S, Zhang Y, Qin L, et al. A role of the CTCF binding site at enhancer Eα in the dynamic chromatin organization of the Tcra-Tcrd locus. Nucleic Acids Res. 2020;48(17):9621-36.

45. Ratiu JJ, Barclay WE, Lin E, Wang Q, Wellford S, Mehta N, et al. Loss of Zfp335 triggers cGAS/STING-dependent apoptosis of post-β selection thymocytes. Nat Commun. 2022;13(1):5901.

46. Naik AK, Byrd AT, Lucander ACK, Krangel MS. Hierarchical assembly and disassembly of a transcriptionally active RAG locus in CD4(+)CD8(+) thymocytes. J Exp Med. 2019;216(1):231-43.

47. Fahl SP, Contreras AV, Verma A, Qiu X, Harly C, Radtke F, et al. The E protein-TCF1 axis controls γδ T cell development and effector fate. Cell Rep. 2021;34(5):108716.

**Supplementary Table 2.** Antibodies

| Antibody | Fluorophore | Clone | Vendor | Cat. No. | RRID |
| --- | --- | --- | --- | --- | --- |
| Anti-TCRδ | PE-CF594 | GL3 | BD Biosciences | 563532 | RRID:AB_2661844 |
| Anti-TCRβ | BV711 | H57-597 | BioLegend | 109243 | RRID:AB_2629564 |
| Anti-CD4 | BV605 | RM4-5 | BioLegend | 100548 | RRID:AB_2563054 |
| Anti-CD8 | BV785 | 53-6.7 | BioLegend | 100750 | RRID:AB_2562610 |
| Anti-CD3 | PE | 17A2 | BioLegend | 100206 | RRID:AB_312663 |
| Anti-CD69 | Kiravia Blue 520 | H1.2F3 | BioLegend | 104554 | RRID:AB_2832335 |
| Anti-CD24 | Pacific Blue | M1/69 | BioLegend | 101820 | RRID:AB_572011 |
| Anti-CD44 | BV650 | IM7 | BioLegend | 103049 | RRID:AB_2562600 |
| Anti-NK1.1 | PE-Cy7 | PK136 | BioLegend | 108714 | RRID:AB_389364 |
| Anti-CD44 | PE-Cy7 | IM7 | BioLegend | 103030 | RRID:AB_830787 |
| Anti-CD3 | FITC | 17A2 | BioLegend | 100204 | RRID:AB_312661 |
| Anti-CD69 | PE | H1.2F3 | BioLegend | 104508 | RRID:AB_313111 |
| Anti-NK1.1 | PE Dazzle 594 | PK136 | BioLegend | 108748 | RRID:AB_2564219 |
| Anti-TCRδ | FITC | GL3 | BioLegend | 118106 | RRID:AB_313830 |
| Anti-TCRβ | APC-Cy7 | H57-597 | BioLegend | 109220 | RRID:AB_893624 |
| Anti-CD11b | PE-Cy5 | M1/70 | BioLegend | 101210 | RRID:AB_312793 |
| Anti-CD11c | PE-Cy5 | N418 | BioLegend | 117316 | RRID:AB_493566 |
| Anti-F4/80 | PE-Cy5 | BM8 | BioLegend | 123112 | RRID:AB_893482 |
| Anti-CD19 | PE-Cy5 | 6D5 | BioLegend | 115510 | RRID:AB_313645 |
| Anti-TER-119 | PE-Cy5 | TER-119 | BioLegend | 116210 | RRID:AB_313711 |
| Anti-CD45R/B220 | PE-Cy5 | RA3-6B2 | BioLegend | 103210 | RRID:AB_312995 |
| Anti-Ly-6G/Ly-6C (gr-1) | PE-Cy5 | RB6-8C5 | BioLegend | 108410 | RRID:AB_313375 |
| Anti-TCRδ | PE-Cy5 | GL3 | Invitrogen | 15-5711-82 | RRID:AB_468804 |
